# Drawing the Line Between Obsessive-Compulsive Disorder and Schizophrenia

**DOI:** 10.7759/cureus.36227

**Published:** 2023-03-16

**Authors:** Tânia B Cavaco, Joana S Ribeiro

**Affiliations:** 1 Child and Adolescent Psychiatry, Garcia de Orta Hospital, Almada, PRT; 2 Psychiatry and Mental Health, Centro Hospitalar Vila Nova de Gaia/Espinho, Vila Nova de Gaia, PRT

**Keywords:** obsessive-compulsive symptoms, schizo-obsessive disorder, obsessive-schizophrenia spectrum, obsessive-compulsive disorder, schizophrenia

## Abstract

The schizo-obsessive spectrum has been a central focus of interest and research within the scientific community in mental health. The increased comorbidity of schizophrenia and obsessive-compulsive symptoms (OCS) or obsessive-compulsive disorder (OCD) appears to be considerably higher than previously expected, with more recent studies suggesting growing prevalence rates. Despite this phenomenon, OCS are not considered primary manifestations of schizophrenia and are therefore not usually explored in these patients. The concept of schizo-obsessiveness mostly emerged in the 1990s, progressing into OCD-schizophrenia spectrum disorders as a dual diagnosis of OCD and schizophrenia. The manifestations of the schizo-obsessive spectrum are diverse, and its diagnoses may be divided overall into four main categories: schizophrenia with OCS; schizotypal personality disorder (SPD) with OCD; OCD with poor insight; schizo-obsessive disorder (SOD). In some cases, distinguishing an intrusive thought from delirium in OCD with poor insight might be challenging. Poor or absent insight can be present in many diagnoses of OCD. Those patients within the schizo-obsessive spectrum present a worse insight than those with OCD without schizophrenia. The comorbidity has important clinical implications, considering its association with an earlier onset of the disorder, more severe positive and negative psychotic symptoms, a greater cognitive deficit, more severe depressive symptoms, more suicide attempts, a reduced social network, increased psychosocial dysfunction, and consequently a worse quality of life and greater psychological suffering. The presence of OCS or OCD in schizophrenia may lead to more severe psychopathology and a worse prognosis. More precise diagnoses allow for a more targeted intervention by offering an optimized psychotherapeutic and psychopharmacological approach. We hereby present four clinical cases that represent each of the four designated categories of the schizo-obsessive spectrum. This case-series report aims to enhance clinical insight regarding the diversity of the schizo-obsessive spectrum and to illustrate the difficult and sometimes misleading process of differentiating OCD from schizophrenia and establishing a diagnosis due to the potential overlap of phenomenology, as well as the course and assessment of symptoms manifested within the spectrum.

## Introduction

The schizo-obsessive spectrum disorders have been an increasing focus of investigation and interest within the scientific community in mental health. The remarkably high comorbidity of schizophrenia and obsessive-compulsive symptoms (OCS) and obsessive-compulsive disorders (OCD) appears to be considerably higher than previously expected [1,2]. The co-occurrence of OCS and schizophrenia has been studied for more than a century, after Westphal presented the first description of OCD in patients with schizophrenia in 1878, later followed by Janet (1903) and Bleuler (1911) [3].

The prevalence rate of schizophrenia in the general population is ~1%, whereas the prevalence of OCD is about 2-3% (9). OCS and OCD appear to co-occur in an important percentage of patients with schizophrenia, with prevalence rates ranging from 10% to 64% for OCS and 7.8% to 31.7% for OCD [4], with more recent studies pointing to 25% for OCS and 12% for OCD [5]. This comorbidity has clinical implications considering its association with poorer quality of life, increased dysfunction, more suicide attempts, more reduced social networks, and, therefore, increased suffering [6]. Hence, the untimely identification of OCS in schizophrenia is of utmost significance regarding the patient’s clinical outcome, especially considering the good clinical response of OCS to treatment.

Evidence suggests that in schizophrenia, obsessive-compulsive phenomena are independent of nuclear psychotic symptoms; however, differentiating obsessions from delusions and even perhaps compulsions from stereotypical behaviour may result in difficulties in clinical practice. The ideas present in obsessive-compulsive symptoms overlap with those in delusions or hallucinations, and therefore, some authors have hypothesised that OC characteristics and those in psychosis might coexist in a psychopathologic complex [7]. Despite the high prevalence of these two entities, OCS are not considered primary manifestations of schizophrenia, and thus these patients are not usually routinely screened for OCS [8]. In fact, up until the 1980s, most OCD patients were being diagnosed as having schizophrenia. When the association between the two disorders became clearer, psychotic-like symptoms appeared to be shared in OCD and were present in different disorders as a common dimension [9].

The concept of "schizo-obsessiveness" was first presented by Hwang and Opler in 1994, the same year in which the fourth edition of the Diagnostic and Statistical Manual of Mental Disorders (DSM-IV) permitted the dual diagnosis of schizophrenia and obsessive-compulsive disorders. Posteriorly, the OCD-schizophrenia spectrum disorders have been further investigated by Poyurovsky and Koran, including OCD, OCD with poor insight, OCD with schizotypal personality disorder (SPD), schizophrenia with obsessive-compulsive symptoms, schizophrenia with OCD, and pure schizophrenia [10]. This led to the establishment of a new entity - the schizo-obsessive disorder (SOD) - in which patients present with OCD along with positive, negative, and cognitive schizophrenia symptoms. OCD is found to be associated with poor or absent insight in 15-30% of OCD diagnoses [11,12], but depending on how the evaluation is elaborated, this fraction might increase to 40% [13].

The following case series represents four cases of young adult psychiatric patients displaying different clinics within the OCD-schizophrenia spectrum: (1) schizophrenia and OCS; (2) SPD associated with OCD; (3) OCD with poor insight; and (4) SOD (dual diagnoses for schizophrenia and OCD). This case series aims to illustrate the difficult and sometimes misleading process of differentiating OCD from schizophrenia, or of establishing a diagnosis, with regard to the onset of symptom presentation, the sometimes overlap of phenomenology, the course and assessment of symptoms, as well as the importance of this differentiation.

## Case presentation

Case 1: Schizophrenia and obsessive-compulsive symptoms

A 26-year-old male, single, lives with his parents, finished high school at the expected age, has applied twice to university but has dropped out, and is currently unemployed. The patient denies any active or past drug consumption. The patient’s first contact with psychiatry was at 19 years old, in 2016, at a psychiatric emergency service, when he was admitted to psychiatric internment for 21 days. At that time, the patient presented with severe depressive symptoms (sadness, isolation, insomnia, thoughts of death, and anhedonia) and obsessive thoughts about the impact of sound pollution on hearing, which the patient refers to as having increased along with the depressive symptoms. Previously to the depressive symptoms, the patient referred to "feeling something strange might have been happening and be about to happen," to which he could not elaborate. This was concurrent with disorganized speech, derailment, and loose association of ideas. The patient also described the phenomena of thought diffusion and blocking without presenting any insight into his medical condition. According to the patient's clinical history, he has had a rigid profile and obsessive contamination- and money-related thoughts since he was a teenager. The patient was medicated with paliperidone 14 mg, od, during his admission and was referred for psychiatric consultations after being discharged. During his outpatient follow-up, the patient presented poor adhesion to medication, and therefore injectable palmitate paliperidone was suggested, which the patient rejected - the patient was resistant to any medication. Due to the maintenance of OCS, fluvoxamine 100 mg, od, was started, with symptomatic improvement, which the patient autonomously ended up suspending. In the following year, therapeutic adhesion to antipsychotic per os was dubious, and the patient occasionally presented with delusional grandiose ideas regarding the future and "a feeling of emptiness and apathy" sic. Elevated mood or maniform symptoms were never identified throughout the patient’s clinical history or follow-up, at any moment. After the remission of psychotic symptoms, the patient presented with a depressed mood, low self-esteem, bradypsychism, passive ideas of death, and affective flattening. Depressive symptoms were never present concurrently with the positive psychotic symptoms and were regarded as a depressive reaction to the primary psychotic episode. Paliperidone was scaled down to 18 mg with the poor clinical benefit regarding psychotic symptoms, and palmitate paliperidone 100 mg, IM, monthly, was then started. A year later, the patient started displaying an increase in negative symptoms and refused injectable medication, being compulsively admitted to the psychiatric ward for 38 days due to developing negative and positive psychotic symptoms, including isolation, defensiveness, psychomotor slowing, paranoia, soliloquies, mystical delusions, and medication refusal, along with an aggravation of OCS manifestation. The patient was diagnosed with schizophrenia, presenting an overall improved status and remission of positive symptoms at discharge despite still presenting important negative symptoms (isolation and social avoidance, occasional soliloquies, and defensiveness). Regardless of the remission of positive symptoms, the presence of moderate OCS was sustained (handwashing, worries about contamination, and verification rituals), which did not greatly interfere with his daily functioning. An increase to 150 mg of palmitate paliperidone monthly was made with improvements regarding both psychotic and OCS symptoms, which appeared to be linked to the psychotic symptoms. Up to this day, the patient presents no insight for his psychotic symptoms and denies needing psychiatric medication; however, he has presented insight for his egodystonic OCS and is currently under treatment with monthly palmitate paliperidone 150 mg, IM, and fluvoxamine 100 mg, od.

Case 2: Schizotypal personality disorder and obsessive-compulsive disorder

A 20-year-old, male, unemployed, single, lives with his mother and brother. The patient studied up to the 10th grade in high school, having dropped out after completing 18 years of age. The patient’s cognitive assessment is unknown; however, the patient’s mother reports worsening in the patient's cognitive function and academic performance since his late adolescence, along with an impulsive and emotionally immature profile. The patient’s pre-morbid functionality was guided by moral rigidity and elevated moral patterns, with a melancholic temperament. The patient was followed up in child psychiatry since he was 13 years old, due to symptoms of moderate anxiety and obsessive, egodystonic thoughts of a sexual and aggressive nature, as well as depressive symptoms, secondary to the OCD. The patient relates the latter to the anguish caused by the OCS, including daily anguish, isolation, sadness, a decrease in interaction and communication, and insomnia, which deeply interfered with his functioning. He was diagnosed with OCD and was started on sertraline 50 mg, od, with mild improvement, which was scaled to sertraline 100 mg, od, with a moderate therapeutic response; however, the patient ended up stopping his medication due to poor adhesion. The intrusiveness of obsessive thoughts led to further isolation from peers and friends. His medication was further enhanced with aripiprazole 5 mg, od, with an improved response regarding his symptoms of anguish, impulsiveness, and intrusiveness of thoughts. At 15 years of age, he attempted suicide by self-defenestration at school. Some months later, the patient reports having started smoking weed and hashish 3-4 times a week, with periods of daily consumption, which he associates with an improvement in obsessive thoughts. At 18 years of age, the patient was admitted to the psychiatric ward presenting a suspicious/paranoid and defensive contact, with unstable and inconsistent behavior, anxiety, disorganized speech, and ideas of grandeur, but without a verbalized or structured delusion. During his 37-day admission, the patient was subjected to the projective test Rorschach, which revealed: "Fragility regarding his psychic structure and connection to reality, difficulties in his interpersonal relationships, reporting discomfort and inadaptability in his contact with the world, which he regards as hostile; intense internal life and activity of thought, and scarce externalization; no indicators of clear psychotic disorganization were observed, evidencing a functioning with schizotypal features." After being discharged with a diagnosis of SPD, the patient was referred to psychiatric appointments and medicated with risperidone 25 mg, IM, fortnightly, and risperidone 3 mg, od; however, he did not attend his appointments and had rejected all his psychiatric medication due to a lack of insight regarding his clinical condition, after which the patient started presenting again with disabling suicidal thoughts and persecutory ideas, obsessive racism- and violence-related thoughts, and severe daily cognitive and somatic anxiety. The patient’s medication was switched to paliperidone palmitate 150 mg, IM, monthly, after which he reported feeling more organized. Despite maintaining less intrusiveness of thoughts, intense egodystonic sexual-related thoughts and more depressive feelings have endured, along with a consequent increase in substance consumption. Due to therapeutic abandonment, escalating behavior, and aggravating impulse control, the patient was again compulsory admitted to the psychiatric ward for 34 days, being medicated with paliperidone palmitate 150 mg IM, monthly, olanzapine 5 mg, od, and sertraline 100 mg, bid, at discharge, and having a diagnosis of SPD and OCD. Soon after being discharged, the patient quickly presented an aggravation of both depressive and obsessive thoughts with an apparent delusional pattern, social anxiety, more depressive symptoms, greater isolation, and paranoia directed at his nuclear family, presenting a partial insight into his clinical condition. Through time, the patient’s functioning has worsened, with a great gradual impact on his functionality. The patient is not willing to finish school, nor does he have any determination to find a job. Currently, the patient is medicated with palmitate paliperidone 150 IM, monthly, and has stopped all oral medication due to non-adherence and fluctuation of symptoms.

Case 3: Obsessive-compulsive disorder with poor insight

A 19-year-old male, a high-school student, single, lives with his mother and grandparents. The patient had his first contact with mental health services at 18 years old, presenting with complaints of obsessive-compulsive symptoms, including intrusive thoughts of contamination and tragic thoughts, after many years of evolution. Secondarily, the patient also presented depressive symptoms, including social isolation and anhedonia, which motivated his search for psychiatric consultations. He was medicated with sertraline 50 mg, od and quetiapine 50 mg, od. The patient was later admitted to the psychiatric emergency services, six months after being on follow-up appointments, presenting with an unstructured mystical and grandiose delusion. De novo symptoms of tachypsychia, hypervigilance, and depersonalization, along with manifestations of psychomotor agitation, anguish, early insomnia, mystical ideas, and self-referential paranoia, had arisen weeks before his admission, after being medicated with methylphenidate due to complaints of inattention. During his 40-day internment, the patient was subjected to a psychological assessment, which resulted in a clinical impression of "incipient psychosis." After being discharged, the patient returned to outpatient psychiatric consultations. His clinical history has revealed symptoms of severe anxiety and OCS (handwashing and verification) since he was 12 years old, which appeared to have exacerbated throughout his teenage years and specifically during the COVID-19 pandemic. For the past two years, the patient has also reported depressive symptoms, including sadness, isolation, insomnia, lack of energy, lack of motivation, and disinterest in daily activities. His anxiety and OCS symptoms increased with time, presenting with periods of apparent paranoia and interfering with his socialization with friends and family and his academic performance. The patient was initially medicated with risperidone 50 mg, IM, fortnightly, without clinical improvement, presenting sustained anguish and severe anxiety symptoms due to the maintenance of obsessive symptoms, namely intrusive ideas of tragic content. This way, escitalopram 20 mg, od, and lorazepam 2.5 mg were initiated while his antipsychotic was suspended, with clinical improvement regarding his anxiety, depression, and OC symptoms, along with his functionality, as the patient managed to finish high school and has enrolled in university.

Case 4: Schizo-obsessive disorder (dual schizophrenia and obsessive-compulsive disorder diagnosis)

A 24-year-old male, single, lives with his mother. The patient was previously an undergraduate student, but he has dropped out of university. The patient first started being followed up in psychiatric appointments at 23 years old due to obsessive-compulsive symptoms, which resulted in a diagnosis of OCD. He reports having an anxious profile since his school years and developing depressive and OC symptoms of verification as a teenager, although he was never assessed by adolescent psychiatry. According to the patient, OCS has worsened with time. After starting psychiatric consultations, the patient was medicated with aripiprazole 15 mg, od, and venlafaxine 75 mg, od, with only moderate improvement. At age 24, the patient was admitted to the psychiatric ward with a mental warrant from his psychiatrist due to severe aggressive behavior towards objects and his mother, which had become more evident in the past three months. This manifestation presented along with greater isolation, a more disorganized speech, persecutory, self-referential, and magical delusions, intrusive thoughts, describing "feeling relief from ruminative thoughts" sic, and also mentioning he was having "a generalized feeling of strangeness" sic, presenting no insight for his morbid condition. According to the patient, verification rituals and obsessive thoughts had been increasing in frequency and intensity for the past six months. During his internment, OCS appeared to decrease along with the blurring of psychotic symptoms, with a good therapeutic response to oral antipsychotics. The patient was discharged after 27 days after being referred to psychiatric outpatient appointments with a diagnosis of schizophrenia and OCD, considering the pseudoneurotic prodrome. During his follow-up, the patient displayed an irregular adherence to medication, with a great impact on his negative and positive symptoms, including isolation, disorganization, difficulty keeping up with academic or professional tasks, changes in perception, a lack of touch with reality, as well as worsening of OCS. Due to the recurrence of OCS, the patient was medicated with sertraline, which he did not adhere to. The patient mentioned suffering from more intensive OCS every time he stopped taking his antipsychotic medication. The patient was started on palmitate paliperidone 100 mg IM, monthly, fluoxetine 20 mg, od, and trazodone 100 mg, od, and reported a marked improvement in OCD symptomatology as well as both positive and negative symptoms.

## Discussion

OCD may be clinically severe and manifest as symptoms that appear psychotic in nature. Furthermore, this distinction may become even more difficult due to the high variability of insight, which may be lacking in OCD patients and therefore hinder the more typical, egodystonic OCS usually present in these patients [14]. This egodystonic vs. egosyntonic differentiation is close to the concept of insight, which helps identify delusional ideas, and therefore patients presenting with obsessions with poor insight, i.e., egosyntonic, could be confused for delusions [3]. Bottas et al. [15] have suggested a list of criteria to recognize obsessions when acute psychosis is present: OCS analogous to symptoms present in pure OCD, according to the DSM-5; the presence of OCS asynchronous of acute psychotic or dellusional episodes; compulsions resulting from obsessions and not from hallucinations or any other psychotic phenomena; re-evaluation of obsessions after an acute psychotic episode when a thought-form disorder is considered; empiric treatment with a selective serotonin reuptake inhibitor (SSRI) to help discriminate between disorders [3]. Moreover, one study by Szmulewicz et al. showed that OCD-only patients also presented more aggressive, somatic, sexual, and contamination-related content in their obsessions in comparison to those diagnosed with comorbid OCD/schizophrenia, which can also be considered when differentiating between symptoms, and consequently diagnosis [16].

In some cases, distinguishing whether an intrusive thought is a delusion in psychosis or an obsession with OCD with poor insight might be challenging [9]. Therefore, the identification of previous and present symptoms is of utmost importance to establish an adequate diagnosis, considering that bizarre, psychotic-like symptoms or even more disorganized, atypical thoughts could constitute an obsession - which despite appearing odd, might be (remotely) possible [17]. When these thoughts, although bizarre, are a manifestation of OCD, they would respond to serotonergic medication, which reinforces the need for and importance of a correct diagnosis. Growing evidence has shown that this varying spectrum from schizophrenia to OCD is highly influenced by the degree of insight. This is supported by the association between SPD and poor insight, which is present even in treated patients. As would be expected, schizo-obsessive patients present with poorer insight than OCD patients undiagnosed with schizophrenia [5].

The differentiation between OCD and schizophrenia may be elaborated based on four main parameters: (1) integration of each disease’s epidemiological and prevalence data; (2) patient’s clinical history and timing of symptom presentation (for example, regarding the peak onset for OCD being adolescence, contrasting with a peak onset for schizophrenia being young adulthood, might help establish the diagnosis); (3) assessment of the patient’s insight; and (4) follow-up to assess disease course and clinical evolution [14].

The link between OCS/OCD and psychosis has been associated with different timings and mechanisms: The emergence of OCS/OCD as an independent process, arising before the onset of psychosis; OCS/OCD regarded as automatisms, arising prior to psychosis, in at-risk mental states of schizophrenia; the concomitant onset of psychotic and OCS/OCD as primary symptoms of psychosis; OCS/OCD arising after the acute psychotic episode in chronic schizophrenia; and the development of OCS/OCD after starting antipsychotics [18]. Regarding the clinics of the schizophrenia-OCD spectrum, diagnoses found within this spectrum might be distributed into four main entities, which were used to illustrate the clinical cases hereby presented: Schizophrenia with OCS (case 1); SPD associated with OCD (case 2); OCD with poor insight (case 3); and schizo-obsessive disorder (case 4).

OCD may be clinically severe and manifest as symptoms that appear psychotic in nature. Furthermore, this distinction may become even more difficult due to the high variability of insight, which may be lacking in OCD patients and therefore hinder the more typical, egodystonic OCS usually present in these patients [14]. This egodystonic vs. egosyntonic differentiation is close to the concept of insight, which helps identify delusional ideas, and therefore patients presenting with obsessions with poor insight, i.e., egosyntonic, could be confused for delusions [3]. Bottas et al. [15] have suggested a list of criteria to recognize obsessions when acute psychosis is present: OCS analogous to symptoms present in pure OCD, according to the DSM-5; the presence of OCS asynchronous of acute psychotic or dellusional episodes; compulsions resulting from obsessions and not from hallucinations or any other psychotic phenomena; re-evaluation of obsessions after an acute psychotic episode when a thought-form disorder is considered; empiric treatment with a SSRI to help discriminate between disorders [3]. Moreover, one study by Szmulewicz et al. showed that OCD-only patients also presented more aggressive, somatic, sexual, and contamination-related content in their obsessions in comparison to those diagnosed with comorbid OCD/schizophrenia, which can also be considered when differentiating between symptoms, and consequently diagnosis [16].

In some cases, distinguishing whether an intrusive thought is a delusion in psychosis or an obsession with OCD with poor insight might be challenging [9]. Therefore, the identification of previous and present symptoms is of utmost importance to establish an adequate diagnosis, considering that bizarre, psychotic-like symptoms or even more disorganized, atypical thoughts could constitute an obsession - which despite appearing odd, might be (remotely) possible [17]. When these thoughts, although bizarre, are a manifestation of OCD, they would respond to serotonergic medication, which reinforces the need for and importance of a correct diagnosis. Growing evidence has shown that this varying spectrum from schizophrenia to OCD is highly influenced by the degree of insight. This is supported by the association between SPD and poor insight, which is present even in treated patients. As would be expected, schizo-obsessive patients present with poorer insight than OCD patients undiagnosed with schizophrenia [5].

The differentiation between OCD and schizophrenia may be elaborated based on four main parameters: (1) integration of each disease’s epidemiological and prevalence data; (2) patient’s clinical history and timing of symptom presentation (for example, regarding the peak onset for OCD being adolescence, contrasting with a peak onset for schizophrenia being young adulthood, might help establish the diagnosis); (3) assessment of the patient’s insight; and (4) follow-up to assess disease course and clinical evolution [14].

The link between OCS/OCD and psychosis has been associated with different timings and mechanisms: The emergence of OCS/OCD as an independent process, arising before the onset of psychosis; OCS/OCD regarded as automatisms, arising prior to psychosis, in at-risk mental states of schizophrenia; the concomitant onset of psychotic and OCS/OCD as primary symptoms of psychosis; OCS/OCD arising after the acute psychotic episode in chronic schizophrenia; and the development of OCS/OCD after starting antipsychotics [18]. Regarding the clinics of the schizophrenia-OCD spectrum, diagnoses found within this spectrum might be distributed into four main entities, which were used to illustrate the clinical cases hereby presented: Schizophrenia with OCS (case 1); SPD associated with OCD (case 2); OCD with poor insight (case 3); and schizo-obsessive disorder (case 4).

Case 1

In schizophrenia with OCS, which is hereby represented by case 1, patients with schizophrenia may display OCS at any time of the course of psychosis - before and independently of schizophrenia, intermittently, in later stages of the disease, or prodromally and then steadily along with the disease. When OCS are present in schizophrenia, symptoms may be linked to the psychotic phenomena (schizo-obsessive concept) or to antipsychotics [5].

Case 2

Represents SPD associated with OCD, where patients may usually present a more severe decay in functioning and in cognitive performance, poor treatment adhesion, more negative and depressive symptoms, greater severity, more comorbidities with other mental disorders (depression, PTSD, bipolar disorder, or substance abuse), poorer insight, and sometimes transformation of obsessions into delusions, resulting in more severe symptoms and a worse overall prognosis in comparison with OCD-only patients [5]. Other clinical characteristics, such as being male and having an earlier onset of OCS, specific phobias, and counting compulsions, translate into an increased possibility of schizotypy [10]. Hoarding has also been described as often being associated with the comorbidity SPD-OCD or as a predictor behavior of schizotypal traits in OCD.

Case 3

The clinical example of "OCD with poor insight" is exemplified by case 3, which is a condition frequently associated with SPD, which often also presents with an earlier onset of disease, a worse clinical outcome regarding the frequency of OCS, increased duration and chronicity of OCD, greater severity, and a weaker response to treatment. OCD with poor insight also presents a greater risk of comorbidities and of developing schizophrenia-spectrum disorders [5,19].

Case 4

This case illustrates the dual diagnosis of schizophrenia and OCD - SOD - whose patients’ clinical profiles may be controversial; however, OCS in these patients appears more intense and tends to emerge as a prodrome of psychosis, and these are hypothesized to have a deleterious role on schizophrenia. One distinguishable feature of SOD - in contrast with SPD associated with OCD and OCD with poor insight - is the level of insight, which is usually moderate to good, as these patients tend to present mild to good insight regarding OCS, similarly to OCD-only patients. Also, comparably to SPD associated with OCD, these patients show an overall poorer quality of life, with an early onset of psychosis, predominantly in men, and more symptoms of anxiety, including panic episodes and phobias, depression, and suicide attempts, along with lower functioning and greater social impairment, including more hostile behavior [5].

## Conclusions

Psychiatric comorbidities in patients with psychotic-like symptoms or schizophrenia have been widely reported in the literature. Even though this association has been extensively described, the relationship between OCD/OCS and schizophrenia/schizotypal personality still comprises a difficult diagnosis and several clinical problems, mainly due to both OCD and schizophrenia being two complex entities presenting a multiplicity of phenotypes and clinical features.

Overall, the presence of OCS/OCD in schizophrenia may lead to greater severity and a worse prognosis. Patients with schizophrenia and OCS/OCD are more prone to present an early onset of psychopathology, to suffer from more severe positive and negative psychotic symptoms, worse depressive symptoms, a greater risk of suicidality, poorer social functioning, more dysfunctional psychosocial features, and greater cognitive impairment. Moreover, the therapeutic approaches, both psychopharmacology and psychotherapy, may differ, reinforcing the importance of establishing a correct diagnosis, which may allow a more optimized intervention for better prognoses.

## References

[1] Achim AM, Maziade M, Raymond E, Olivier D, Mérette C, Roy MA (2011). How prevalent are anxiety disorders in schizophrenia? A meta-analysis and critical review on a significant association. Schizophr Bull.

[2] Buckley PF, Miller BJ, Lehrer DS, Castle DJ (2009). Psychiatric comorbidities and schizophrenia. Schizophr Bull.

[3] Tezenas du Montcel C, Pelissolo A, Schürhoff F, Pignon B (2019). Obsessive-compulsive symptoms in schizophrenia: an up-to-date review of literature. Curr Psychiatry Rep.

[4] de Haan L, Sterk B, Wouters L, Linszen DH (2013). The 5-year course of obsessive-compulsive symptoms and obsessive-compulsive disorder in first-episode schizophrenia and related disorders. Schizophr Bull.

[5] Scotti-Muzzi E, Saide OL (2017). Schizo-obsessive spectrum disorders: an update. CNS Spectr.

[6] Lysaker PH, Whitney KA (2009). Obsessive-compulsive symptoms in schizophrenia: prevalence, correlates and treatment. Expert Rev Neurother.

[7] Ozdemir O, Tükel R, Türksoy N, Uçok A (2003). Clinical characteristics in obsessive-compulsive disorder with schizophrenia. Compr Psychiatry.

[8] Grover S, Sahoo S, Surendran I (2019). Obsessive-compulsive symptoms in schizophrenia: a review. Acta Neuropsychiatr.

[9] Palermo S, Marazziti D, Baroni S, Barberi FM, Mucci F (2020). The relationships between obsessive-compulsive disorder and psychosis: an unresolved issue. Clin Neuropsychiatry.

[10] Poyurovsky M, Koran LM (2005). Obsessive-compulsive disorder (OCD) with schizotypy vs. schizophrenia with OCD: diagnostic dilemmas and therapeutic implications. J Psychiatr Res.

[11] Alonso P, Menchón JM, Segalàs C (2008). Clinical implications of insight assessment in obsessive-compulsive disorder. Compr Psychiatry.

[12] Catapano F, Sperandeo R, Perris F, Lanzaro M, Maj M (2001). Insight and resistance in patients with obsessive-compulsive disorder. Psychopathology.

[13] Aigner M, Zitterl W, Prayer D (2005). Magnetic resonance imaging in patients with obsessive-compulsive disorder with good versus poor insight. Psychiatry Res.

[14] Duarte-Mangas M, Bravo L, Matos-Pires A (2020). When obsessive-compulsive disorder mimics schizophrenia. J Nerv Ment Dis.

[15] Bottas A, Cooke RG, Richter MA (2005). Comorbidity and pathophysiology of obsessive-compulsive disorder in schizophrenia: Is there evidence for a schizo-obsessive subtype of schizophrenia?. J Psychiatry Neurosci.

[16] Szmulewicz AG, Smith JM, Valerio MP (2015). Suicidality in clozapine-treated patients with schizophrenia: role of obsessive-compulsive symptoms. Psychiatry Res.

[17] Morgado P (2015). Is "plausibility" a core feature of obsessions?. Braz J Psychiatry.

[18] Schirmbeck F, Zink M (2013). Comorbid obsessive-compulsive symptoms in schizophrenia: contributions of pharmacological and genetic factors. Front Pharmacol.

[19] Tumkaya S, Karadag F, Oguzhanoglu NK, Tekkanat C, Varma G, Ozdel O, Ateşçi F (2009). Schizophrenia with obsessive-compulsive disorder and obsessive-compulsive disorder with poor insight: a neuropsychological comparison. Psychiatry Res.

